# Development and Validation of an Artificial Intelligence-Based Model to Predict Gastroesophageal Reflux Disease After Sleeve Gastrectomy

**DOI:** 10.1007/s11695-022-06112-x

**Published:** 2022-05-21

**Authors:** Sameh Hany Emile, Waleed Ghareeb, Hossam Elfeki, Mohamed El Sorogy, Amgad Fouad, Mohamed Elrefai

**Affiliations:** 1 General Surgery Department, Mansoura University Hospitals, Mansoura University, Mansoura, Egypt; 2 Gastrointestinal Surgery Unit, Department of Surgery, Faculty of Medicine, Suez Canal University Hospital, Ismailia, Egypt; 3 Gastrointestinal Surgery Centre, Mansoura University, Mansoura, Egypt

**Keywords:** Artificial intelligence, Sleeve gastrectomy, Gastroesophageal reflux disease, Predict, Model

## Abstract

**Purpose:**

Prediction of the onset of de novo gastroesophageal reflux disease (GERD) after sleeve gastrectomy (SG) would be helpful in decision-making and selection of the optimal bariatric procedure for every patient. The present study aimed to develop an artificial intelligence (AI)-based model to predict the onset of GERD after SG to help clinicians and surgeons in decision-making.

**Materials and Methods:**

A prospectively maintained database of patients with severe obesity who underwent SG was used for the development of the AI model using all the available data points. The dataset was arbitrarily split into two parts: 70% for training and 30% for testing. Then ranking of the variables was performed in two steps. Different learning algorithms were used, and the best model that showed maximum performance was selected for the further steps of machine learning. A multitask AI platform was used to determine the cutoff points for the top numerical predictors of GERD.

**Results:**

In total, 441 patients (76.2% female) of a mean age of 43.7 ± 10 years were included. The ensemble model outperformed the other models. The model achieved an AUC of 0.93 (95%CI 0.88–0.99), sensitivity of 79.2% (95% CI 57.9–92.9%), and specificity of 86.1% (95%CI 70.5–95.3%). The top five ranked predictors were age, weight, preoperative GERD, size of orogastric tube, and distance of first stapler firing from the pylorus.

**Conclusion:**

An AI-based model for the prediction of GERD after SG was developed. The model had excellent accuracy, yet a moderate sensitivity and specificity. Further prospective multicenter trials are needed to externally validate the model developed.

**Graphical Abstract:**

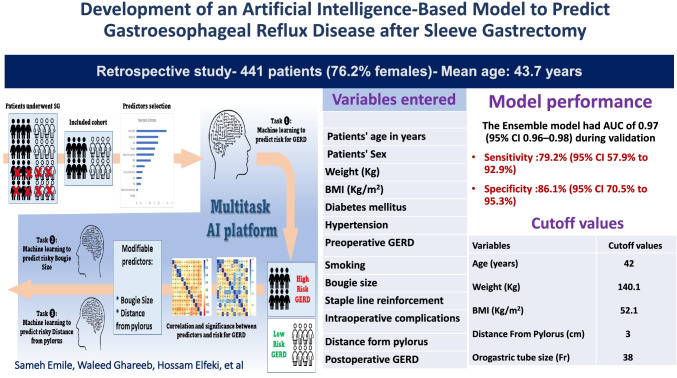

## Introduction

Sleeve gastrectomy (SG) is the most commonly performed bariatric procedure across the world [1]. The popularity of SG has emanated from the excellent results attained by the procedure in terms of weight loss and improvement in comorbidities, in addition to its good safety profile [2]. However, akin to every other surgical procedure, SG is associated with a number of adverse events, including staple line leak and bleeding, gastric stenosis, volvulus, and gastroesophageal reflux disease (GERD).

A recent meta-analysis of 46 studies comprising more than 10,000 patients found that the long-term prevalence of GERD and Barrett’s esophagus after SG is 28% and 8%, respectively [3]. Moreover, 4% of patients may need conversion from SG to Roux-en-Y gastric bypass (RYGB) to treat severe reflux esophagitis. Several hypotheses have been suggested to explain post-SG GERD, and they include increased intraluminal pressure, loss of gastric compliance, removal of the gastric fundus, decreased lower esophageal sphincter (LES) pressure, twisting of the pouch, and persistence of hiatal hernia [4]. In contradiction, other reports documented an improvement in preoperative GERD after SG [5, 6]. Mechanisms involved in the relief of GERD after SG may include reduction of acid production, decrease in intra-abdominal pressure, reduction of gastric volume, and acceleration of gastric emptying [4].

These conflicting findings stimulated further investigation on the impact of SG on the anatomic and physiologic function of the gastroesophageal junction. A recent prospective study [7] concluded a significant decrease in the resting LES pressure after SG in patients without preoperative GERD, yet not in patients with symptomatic GERD. Also, esophageal manometry seemed to have a limited role in the detection of GERD after SG, unlike 24-h pH monitoring which could clearly identify improvement or persistence of GERD.

The prediction of the onset of de novo GERD after SG has become an utmost need to help guide decision-making and selection of the optimal bariatric procedure for every patient [8]. A study including 213 patients found that higher preoperative BMI was less likely to be associated with new-onset or worsening of GERD symptoms whereas more severe heartburn symptoms on standing were predictive of increased risks of developing or worsening GERD symptoms [9].

It has been assumed that if GERD can be predicted before SG, it would guide clinicians to either select another less refluxogenic procedure such as RYGB or to counsel the patients about the need to do frequent upper GI endoscopy after SG, should they still want to have the procedure. Therefore, the present study aimed to develop an artificial intelligence (AI)-based model to predict the onset of GERD after SG to help clinicians and surgeons in decision making.

## Patients and Methods

### Study Design and Setting

This was a retrospective review of prospectively maintained data on patients with severe obesity who underwent SG in the period of January 2011 through December 2019. Patients had their surgical treatment in the General Surgery Department and Gastrointestinal Surgery Center of Mansoura University. Ethical approval for the study was obtained from the institutional review board (IRB) of Mansoura Faculty of Medicine and the study was conducted in compliance with the ethical principles of the Declaration of Helsinki.

### Patient Population

The study enrolled adult patients of either sex with severe obesity defined as BMI > 40 kg/m^2^ or > 35 kg/m2 with at least one associated major comorbidity who underwent laparoscopic SG (LSG). Patients with secondary obesity due to endocrine disorders and those who underwent revisional bariatric procedures or bariatric operations other than SG were excluded. Patients with missing data on the outcome of SG and preoperative and postoperative GERD were also excluded.

### Technique of Sleeve Gastrectomy

All procedures were done under general anesthesia with the patients placed in the French position. Procedures were performed or supervised by expert laparoscopic and bariatric surgeons. The procedure was done in a standardized stepwise manner as follows.

Pneumoperitoneum at 12 mm-Hg was created; then a standard four-port technique was used. Harmonic ace scalpel ™ was used to dissect the greater omentum off the greater.

curvature, beginning 2–6 cm away from the pylorus and proceeding up to the angle of His. Then, an orogastric tube (size of 33, 36, 38, or 42 Fr) was placed in the stomach for calibration followed by transection of the stomach using linear staplers, starting 2–6 cm from the pylorus up to the.

angle of Hiss. Upon creation of the gastric pouch, hemostasis was ascertained; then an intraperitoneal drain was placed before deflation of the abdomen. Hiatal defects were repaired routinely with suture cruroplasty during SG.

### Assessments

Patients were assessed preoperatively with routine laboratory investigations, EKG, chest X-ray, upper GI endoscopy, and pulmonary function tests. Postoperatively, patients were assessed at one and two weeks then at 3, 6, and 12 months postoperatively. Follow-up upper GI endoscopy was performed as part of routine follow-up at 6 and 12 months after SG. Patients with symptoms of GERD before were assessed using the GERD-Q score [10]. A total score of 0 to 2 points implied 0% likelihood of GERD; 3 to 7 points implied 50% likelihood; 8 to 10 points implied 79% likelihood; 11 to 18 points implied 89% likelihood. Patients with a score greater than 2 (≥ 50% likelihood of GERD) or otherwise with a known history of GERD were investigated with upper GI endoscopy.

Endoscopy identified GERD as single or multiple (circumferential or non-circumferential) erosions above the gastroesophageal junction and graded it according to the Savary-Miller classification into four grades. Patients with confirmed GERD were under proton pump inhibitor treatment. Weight loss was expressed as %total weight loss (TWL) and was calculated as [(preoperative weight − weight on follow-up)/preoperative weight] × 100.

### Data Collected

The following data were extracted from patients’ records:

- Demographic data: age, sex, weight, BMI, and smoking status
- Medical comorbidities: diabetes mellitus (DM), hypertension, hyperlipidemia, sleep apnea, and preoperative GERD
- Technical details: size of orogastric calibration tube, distance of transection from the pylorus, staple line reinforcement, and intraoperative complications
- Weight loss at 6 and 12 months after SG
- Postoperative GERD as confirmed by upper GI endoscopy

### Development of the AI-Based Model

Using the available data points, an AI-based model will be developed employing machine learning algorithms through the following steps.

#### Data Preprocessing

Patients’ data were refined to make machine learning possible. The dataset was arbitrarily split into two parts: 70% for training and 30% for testing. The training dataset was validated five times to avoid the overfitting of the model and was randomly divided into five parts throughout the validation process, four parts for learning and one for validation.

#### Variable Selection for Model Creation

All variables were ranked and queued based on their importance to make the final prediction. Testing the estimate levels of importance aimed to minimize redundant variables while keeping the maximum relevance to the proposed prediction. Due to data type heterogeneity (numerical and categorical variables), ranking of the variables was performed in two steps: the first step was done using the interaction test algorithm while the second step was permuting out-of-bag observations (variables). The permuted variables estimates were not biased toward numerical predictors (which contain many levels). Estimates of greater importance indicated more important predictors. For accurate selection of important predictors, estimating the predictor importance by permuting out-of-bag observations was compared to the estimates obtained by summing gains in the mean squared error (MSE).

#### Machine Learning Process

Different learning algorithms were used, and the best model that showed maximum performance was selected for the further steps of machine learning. The performance was judged based on the accuracy rate and the area under the receiver operator curve (AUC, ROC). Furthermore, the developed model underwent an optimization process to minimize the predicting error. The desktop application for the GERD prediction AI model can be downloaded from the following link: https://www.dropbox.com/sh/z1nqd7jtpmp97z2/AADhgez0bYtkh-OqixSl0MMca?dl=0.

### Statistical Analysis

Analysis of data was conducted using SPSS program (version 23, IBM) for Windows. Continuous variables were described as mean and standard deviation (SD) or median and normal range if data were not normally distributed. Student’s *t* test was used to compare continuous variables. Categorical variables were expressed as numbers and proportions and were compared using Chi-square or Fisher exact test. A *p* value less than 0.05 was considered significant.

## Results

### Characteristics of Patients

The present study included 441 patients of a mean age of 43.7 ± 10 years. Patients were 336 (76.2%) female and 105 (23.8%) male. The mean weight of patients was 136.3 ± 25.1 kg, and the mean BMI was 50.7 ± 7.7 kg/m^2^. In total, 44 (9.9%) patients had DM, 77 (17.5%) had hypertension, 95 (21.5%) had sleep apnea, and 35 (7.9%) had hyperlipidemia. Nine patients were smokers (Table 1).

**Table 1:** Characteristics of the cohort studies

Parameter	Value
Age	43.7 ± 10
Male/female	105/336
Mean weight in kg	136.3 ± 25.1
Mean body mass index in kg/m^2^	50.7 ± 7.7
Comorbidities	
Diabetes mellitus Hypertension Hyperlipidemia Sleep apnea GERD	44 (9.9%) 77 (17.5%) 35 (7.9%) 95 (21.5%) 91 (20.6%)
Smokers	9

A total of 91 (20.6%) patients had preoperative GERD with a median GERD-Q score of 9 (range, 3–16). By endoscopic assessment, 84.6% of patients had grade I GERD, 11% had grade II GERD, and 4.4% had grade III GERD. A total of 18 patients had evidence of hiatus hernia in endoscopy and intraoperatively.

### Technical Details

A 33-F orogastric tube was used in 4 (0.9%) patients, 36 F in 184 (41.7%) patients, 38 F in 230 (52.1%) patients, and 42 F in one (0.2%) patient. Staple line reinforcement was done in 14 (3.2%) patients. The distance of starting gastric transection from the pylorus varied from 2 cm (123 patients), 3 cm (14 patients), 4 cm (194 patients), 5 cm (13 patients), and 6 cm (58 patients).

### Weight Loss and Postoperative GERD

BMI decreased significantly from a baseline of 50.7 ± 7.7 to 34.1 ± 4.6 kg/m^2^ at 12 months after SG. The mean %TWL at 12 months was 30.4 ± 6.9%.

Among 91 patients with preoperative GERD, 50 (55%) showed improvement or resolution in GERD after SG whereas 41 (45%) had persistent or worsening GERD symptoms. In addition, 46 additional patients developed new-onset GERD after SG, amounting to a total of 87 (19.7%) patients who had postoperative GERD (49.4% grade I, 38% grade II, and 11.5% grade III).

### Selection of GERD Predictors

**Fig. 1:**
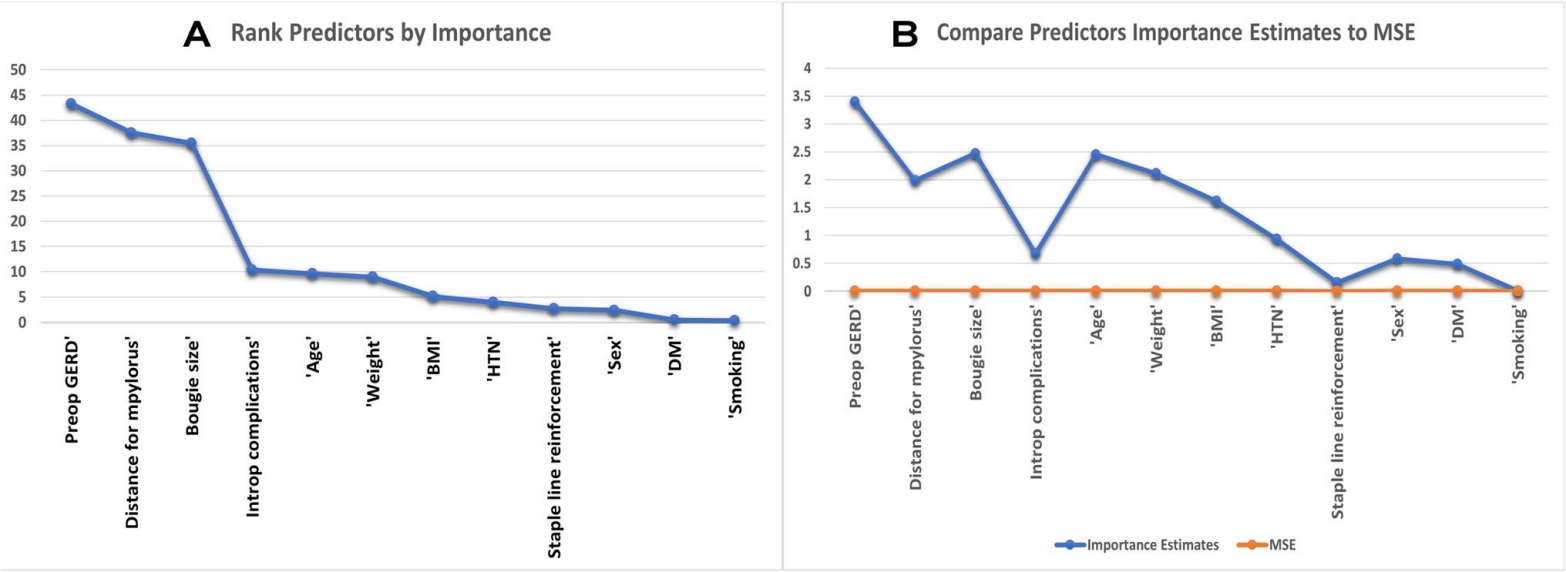
**A** Ranking predictors by importance; **B** comparing predictor importance estimates

The study cohort has been divided into 90% for machine learning set (training set = 70% and validation set = 20%) and 10% for testing set. There was no statistical difference between the input predicting variables of the two sets (Table 2). Predictor selection minimizes the amount of data used in machine learning by choosing only the most important predictors/variables that give the best accuracy for the prediction process. The included predictors were ranked by importance to predict GERD after SG. As an initial step to check for predictors’ importance, all predictors were deemed important, and none was excluded. Preoperative GERD, distance from the pylorus, orogastric tube size, intraoperative complication, and age were ranked as the top five important predictors (Fig. 1A). Since there were two types of predictor types (continuous and categorical), a further algorithmic assessment was required to avoid bias of predictor selection by comparing the importance estimates to the mean squared error (MSE). Although all predictors remained important for the accurate prediction of GERD, the rank of predictors was permuted by importance (e.g., preoperative GERD, bougie size, age, weight, and distance from the pylorus) (Fig. 1B).

**Table 2:** Predictive variables of the machine learning and testing sets used in building predictive models for postoperative gastroesophageal reflux disease

Predictors	Machine learning set	Testing set	*p* value
Patients’ age in years, mean (SD)	34.6 (9.8)	36.1 (11.7)	0.35
Patients’ sex (%)			
Male Female	94 (23.7) 303 (76.3)	11 (25) 33 (75)	0.85
Weight (*Kg*), mean (SD)	135.9 (34.5)	140.1 (30.1)	0.31
BMI (*Kg/m*^*2*^), mean (SD)	50.7 (7.6)	51.3 (9.4)	0.64
Diabetes mellitus (%)			
Yes No	41 (10.3) 356 (89.7)	3 (10) 41 (90)	0.46
Hypertension (%)			
Yes No	66 (16.6) 331 (83.4)	11 (17.5) 33 (82.5)	0.17
Preop GERD (%)			
Yes No	83 (20.9) 313 (79.1)	8 (18.2) 36 (81.8)	0.74
Smoking (%)			
Yes No	8 (2) 389 (98)	1 (2.3) 43 (97.7)	0.91
Bougie size			
33 F 36 F 38 F 42 F	4(1.1) 168 (44.4) 205 (54.2) 1 (0.3)	0 16 (39) 25 (61) 0	0.78
Staple line reinforcement (%)			
Yes No	13 (3.3) 384 (96.7)	1 (2.3) 43 (97.7)	0.72
Intraoperative complications (%)			
Yes No	52 (13.1) 345 (86.9)	5 (11.3) 39 (88.7)	0.75
Distance from the pylorus in cm, Mean (SD)	3.7 (1.3)	3.6 (1.5)	0.91
Postoperative GERD (%)			
Yes No	79 (19.9) 318 (80.1)	8 (18.2) 36 (81.8)	0.79

BMI, body mass index; GERD, gastroesophageal reflux disease

### Machine Learning and Model Optimization

Five models were trained using 15 different algorithms. The ensemble model trained by the bagged algorithm outperformed the 15 models trained by different algorithms (Table 3). To ensure the quality of model performance, the ensemble model was tuned by a hyperparameter optimization process which was continued until reaching the slightest minimum classification error (0.097).

**Table 3:** Predictive performance of different models trained on different algorithms

Algorithm	AUC	Sensitivity (%)	Specificity (%)	PPV (%)	NPV (%)
Decision tree					
*Fine* *Medium* *Coarse*	0.84 0.8 0.73	78.48 60.09 85.45	78.93 84.28 70.07	78.83 79.26 74.06	78.57 67.86 82.81
Logistic regression	0.76	68.57	69.62	69.30	68.90
Naïve Bayes					
*Gaussian* *Kemel*	0.75 0.74	65.92 69.51	70.75 66.04	69.27 67.18	67.49 68.41
SVM					
*Linear* *Quadratic* *Cubic* *Fine* *Medium* *Coarse*	0.73 0.8 0.85 0.87 0.8 0.76	43.50 57.40 70.85 73.09 47.09 45.74	87.42 84.28 80.82 89.94 93.08 89.94	77.57 78.50 78.69 87.90 87.19 81.97	60.74 66.42 73.49 76.97 63.76 62.37
Ensemble					
*Boosted* *Bagged* *RUSBoosted*	0.85 0.96 0.86	73.09 95.52 76.68	83.33 86.48 77.67	81.43 87.60 77.45	75.59 95.07 76.91

PPV, positive predictive value; NPV, negative predic0tive value, POD, postoperative day. ^*^Statistically significant different AUC from the reference diagonal line at *p* value < 0.05

### Visualization of Prediction/Decision-Making Process

Parallel coordinate plotting was used to visualize the predictors while contributing to the prediction/decision-making process. The predictors were arranged into descending order depending on the importance estimates shown in Fig. 1B. The parallel coordinates plot helped know how the model thinks while taking every input predictor until making its final decision. While the plot showed that the model failed to predict GERD when interpreting the first six important input predictors, inclusion of further predictors served to improve the accuracy of the model confusion.

### Performance Assessment

**Fig. 2:**
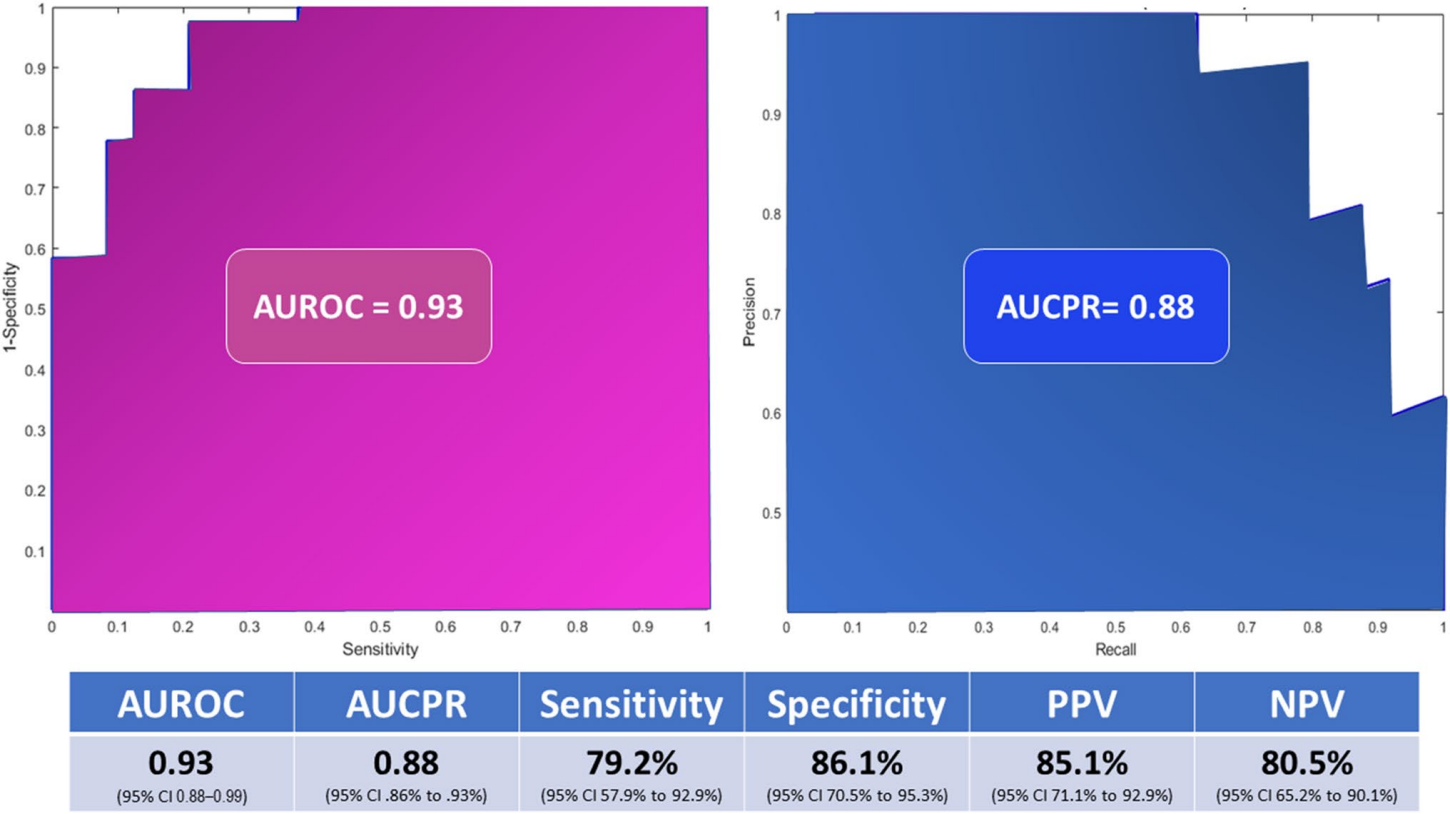
Diagnostic accuracy parameters of the AI model developed

The ensemble model achieved an AUC of 0.97 (95% CI 0.96–0.98) during the validation process. On assessing the performance on the testing dataset, the model achieved an AUC of 0.93 (95% CI 0.88–0.99) and AUCPR of 0.88 (95% CI 0.86% to 0.93%). The model had a sensitivity of 79.2% (95% CI 57.9% to 92.9%), specificity of 86.1% (95% CI 70.5% to 95.3%), PPV of 85.1% (95% CI 71.1% to 92.9%), and NPV of 80.5% (95% CI 65.2% to 90.1%) (Fig. 2).

### Identifying Cutoff Values of GERD Predictors Using a Multitask AI Platform

**Fig. 3:**
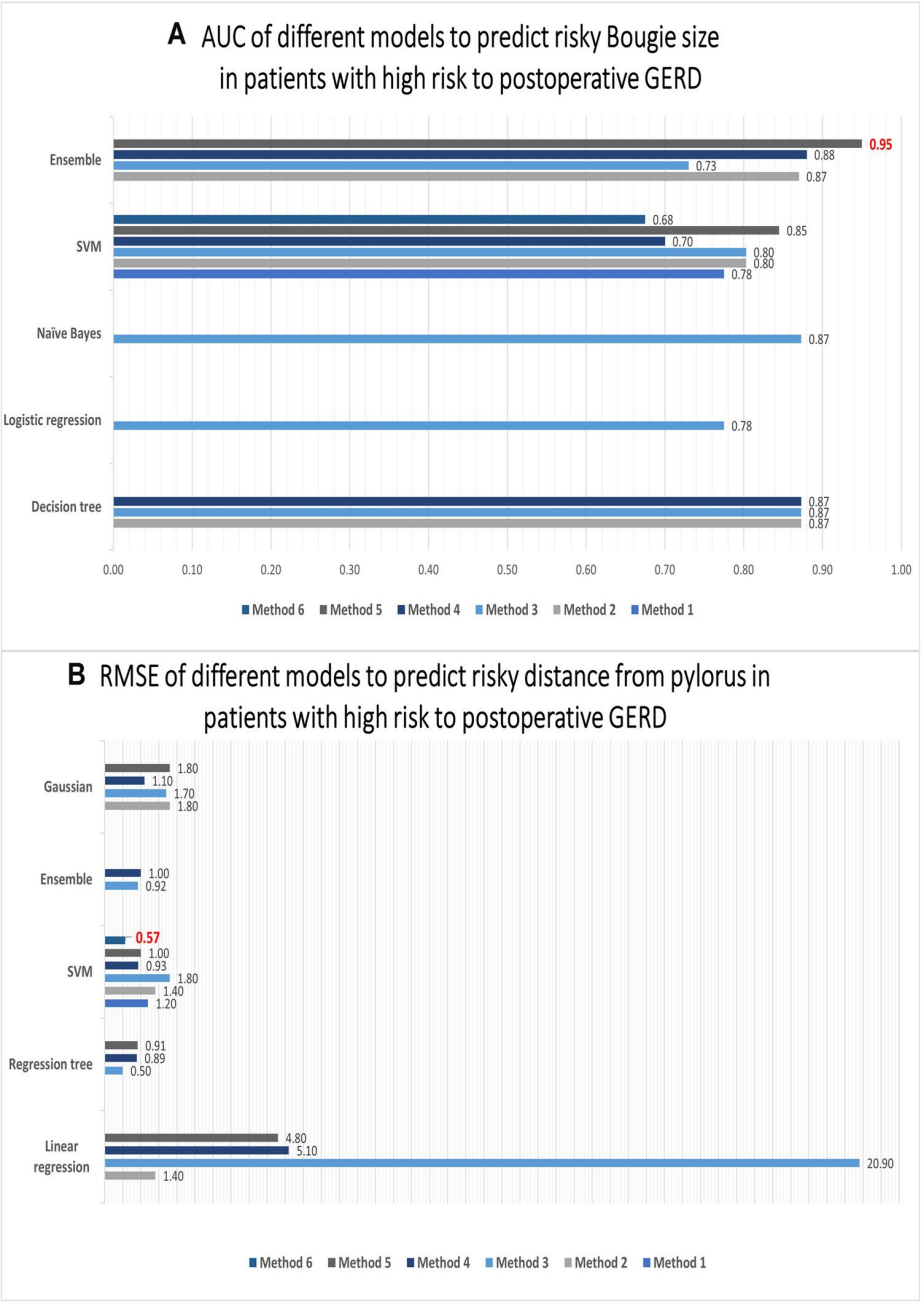
Accuracy of different models used to determine which orogastric tube size and distance from the pylorus are associated with the greatest risk of gastroesophageal reflux disease after sleeve gastrectomy

After the prediction of risk to develop GERD after SG as the first task of the AI platform, patients who were classified as at high risk to develop GERD were included in the next step. The orogastric tube size and distance from the pylorus were two modifiable predictors and were significantly correlated to the risk of developing GERD (*p* = 0.001, 0.02. respectively). Out of 15 trained models with different algorithms, the ensemble model trained by the RUSBoosted method showed the best performance (AU = 0.95) (Fig. 3A). The model predicted the orogastric size to be associated with a higher risk of GERD with an AUC of 0.9 (95% CI 0.86–0.94) and AUCPR of 0.76 (95% CI 0.71% to 0.83%). The third task of the AI platform was achieved by the support vector machine (SVM) model that showed the lowest root mean square error (RMSE 0.57) during machine learning using the coarse Gaussian method (Fig. 3B). The model predicted the distance from the pylorus associated with a higher risk of GERD in the testing dataset with RMSE of 0.81.

The risk of developing postoperative GERD was remarkably increased at the age > 42 years, weight > 140.1 kg, BMI > 52.1 kg/m^2^, orogastric tube < 38 Fr, and distance from the pylorus < 3 cm (odds ratio 1.03, 1.02, 1.1, 1.25, respectively) (Table 4).

**Table 4:** Cutoff values and odds ratio of numerical predictors

Variables	Odds ratio	95% CI	Cutoff values
Age (*years*) Weight (*Kg*) BMI (*Kg/m*^*2*^) Distance from the pylorus (*cm*) Orogastric tube size *(Fr)*	1.03 1.02 1.1 1.25 1.74	0.94–0.99 0.99–1.04 0.86–0.98 0.66–0.98 1.39 -2.18	42 140.1 52.1 3 38

A Sankey diagram illustrating how the surgeon’s decision in choosing the bougie size and the distance from the pylorus can increase or decrease the risk of postoperative GERD in patients who have or do not have preoperative GERD can be accessed through this link file:///C:/Users/DELL/Downloads/Sankey.html.

**Fig. 4:**
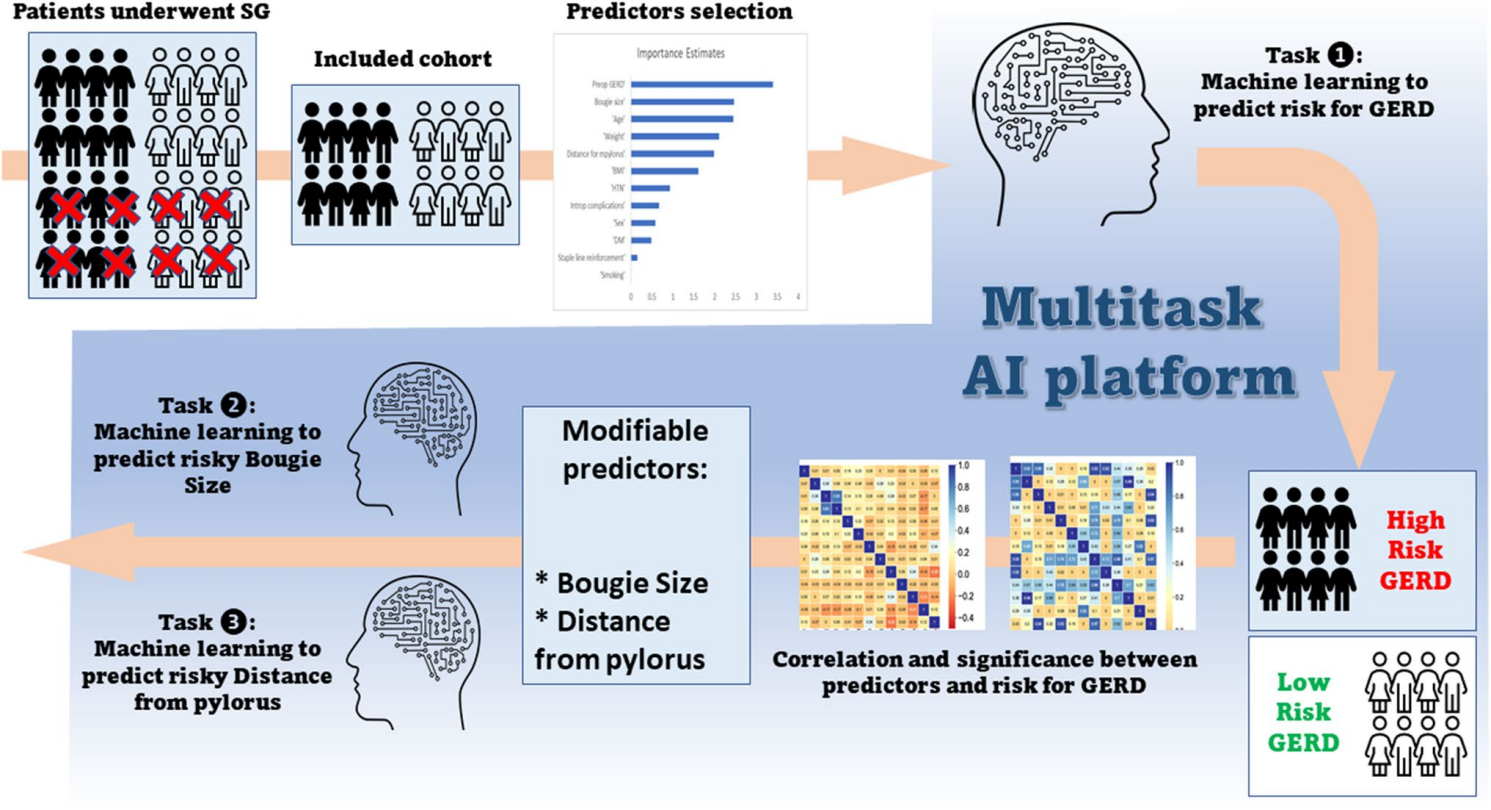
Visual summary of the process of predictor selection and development of the model

A summary of the overall process of predictor selection and model development is shown in Fig. 4.

## Discussion

SG has proven effective in the treatment of severe obesity and its associated comorbidities; however, this may come at the cost of increased risk of adverse events, particularly GERD. It has been estimated that SG may increase the odds of development of new-onset GERD by five times, as compared to other bariatric procedures such as RYGB [11]. This increased risk of GERD may also be associated with more grave consequences such as the development of Barrett’s esophagus and potential onset of adenocarcinoma [3, 12].

Although there is a growing body of evidence that affirms the refluxogenic nature of SG, some reports documented an improvement in pre-existing GERD after SG [4]. Such a controversy implies different mechanisms by which SG impacts the gastroesophageal junction physiology and gastric compliance. Therefore, it became necessary to predict which patients are more liable to develop new-onset GERD after SG which will help counsel these patients about this risk and serve to guide clinicians in choosing alternative procedures.

A number of predictive models for GERD have been described in the literature. A multivariate analysis model found that the visual detection of esophagitis on endoscopy and biopsy-proven esophagitis were strong independent predictors of GERD after SG. Moreover, female patients were more likely to develop GERD postoperatively as compared to male patients [13]. Another study assessed the predictive factors of GERD after bariatric surgery, comparing SG with RYGB. The study found that SG itself was the strongest independent predictor of de-novo GERD and that age, preoperative esophagitis, and postoperative hiatal hernia also independently predicted the onset of GERD after SG [14]. Further evidence was generated from a study [15] that included 213 patients who underwent SG, 47% of whom developed new-onset heartburn after SG. Patients with a higher baseline BMI had lower odds to develop new-onset or worsening symptoms of GERD while more severe heartburn symptoms on standing were associated with higher odds of GERD development (*OR* = 1.22).

It is worthy to note that all the previous predictive models were statistical models, based on multivariate logistic regression analyses. Although this type of models may be able to provide useful data to guide practice, it is limited by the need for a large sample of data to avoid high standard errors in order to provide meaningful results [16]. Moreover, using the stepwise approach for multivariable regression may result in instability of the model and renders the model sensitive to slight changes in data such that addition or omission of a few observations can drastically alter the model [17].

The present model is based on a machine-learning AI model, the use of this kind of modeling has expanded widely in the last few years. AI has been used in a multitude of medical and surgical indications, including the differentiation of benign and malignant skin lesions, predicting colorectal anastomotic leak, and prediction of surgical complications in patients undergoing major abdominal surgery [18–20]. Recently, our group developed an AI-based model that was able to predict acute appendicitis with a sensitivity and accuracy that surpassed the traditional Alvarado scoring system [21].

Based on the initial promising results of AI-based predictive models, we decided to integrate baseline data of patients undergoing SG into an AI model, aiming to help predict the onset of GERD after surgery. Despite the average sensitivity of the model developed by our study (79.2%), the model had an excellent accuracy with an AUC of 97%. Hopefully, with further training and expanded learning process, the sensitivity of the AI model would increase.

Although the AI model included all the available baseline parameters, the top five ranked variables were the presence of baseline GERD, age, weight, size of orogastric tube, and distance of first stapler firing from the pylorus. These parameters, when present, would predictably impact the likelihood of postoperative GERD substantially. Age and BMI have been already recognized as independent predictors of post-SG GERD in previous studies [14, 15]. The presence of baseline GERD symptoms has been also identified as a strong predictor of postoperative GERD [22]. The AI model could recognize the three patient-related parameters among the top predictors of GERD. While older age may be expected to be associated with higher odds of postoperative GERD owing to the documented effects of aging on esophageal and esophagogastric junction mechanophysical properties [23], the association between baseline BMI and postoperative GERD seems to be contentious. Higher baseline BMI could be associated with a higher risk of GERD as it is independently associated with increased intragastric pressure which in consequence may cause an increase in the gastroesophageal-pressure gradient during inspiration, as de Vries and coworkers explained [24]. In contrast, another study [15] found higher baseline BMI to predict a lower incidence of GERD after SG. However, as the authors acknowledged, this finding should be interpreted with caution owing to the small magnitude of effects observed.

Two technical factors were among the top predictors of postoperative GERD as per the developed AI model. The size of orogastric tube reflects the size of the remaining gastric pouch, and with a smaller tube size, a smaller pouch would be constructed. It has been noted that smaller pouches are associated with a higher intraluminal pressure which eventually increases the risk of GERD. Furthermore, increased intragastric pressure was also linked to a higher incidence of staple line leak and GERD, and thus larger bougie size was thought to decrease the risk of leak and GERD [25, 26]. An expert panel consensus [27] concluded that the use of a smaller bougie might be associated with a higher risk of leak, GERD, and other complications. However, a large database study showed a variable impact of the bougie size on the incidence of GERD after SG as larger bougies (≥ 36 F) had a GERD incidence of 28.8–30.7% whereas smaller bougies (< 36 F) had an incidence of 27.5–33.5% [28].

The other technical factor was the distance of the first stapler firing from the pylorus. A meta-analysis [29] found no significant association between the distance from the pylorus and incidence of GERD after SG. However, one main limitation of this meta-analysis was the substantial statistical heterogeneity of the studies included. Interestingly, as shown in this meta-analysis, the odds of developing GERD increased from 1.66 for a distance of 6 cm to 4.79 for a distance of 2–3 cm. This may also be explained on the basis of the intraluminal pressure theory. When the first stapler firing is closer to the pylorus, this would create a narrower gastric tube than if it was farther from the pylorus. Again, with a narrower gastric tube, the intraluminal pressure would be higher and thus increases the risk of GERD.

To expand on the clinical utility of the AI model we developed, we opted to develop a multitask platform that would help in decision-making. This platform identified the cutoff values for age, weight, bougie size, and distance from the pylorus which may help surgeons tailor the modifiable technical factors to reduce the risk of GERD after SG in patients with non-modifiable risk factors as older age and heavier weight.

The limitations of the present study include its retrospective and single-center nature that would be associated with an inherent risk of selection bias and lack of external validity of the results. Larger, multicenter prospective trials are needed to verify the findings of our study and to externally validate the AI model that was developed.

## Conclusion

Using patient-related and technical parameters, an AI-based model for the prediction of GERD after SG was developed. The model had excellent accuracy with an AUC of 0.93, yet a moderate sensitivity of 79.2% and specificity of 86.1%. The top-ranked parameters of the AI model were age, baseline weight, preoperative GERD, size of the orogastric tube, and distance of first stapler firing from the pylorus.
